# Intramedullary versus extramedullary alignment of the tibial component in the Triathlon knee

**DOI:** 10.1186/1749-799X-6-44

**Published:** 2011-08-20

**Authors:** James P Cashman, Fiona L Carty, Keith Synnott, Paddy J Kenny

**Affiliations:** 1 Department of Orthopaedics, Cappagh National Orthopaedic Hospital, Finglas, Dublin 13, Ireland; 2 Department of Radiology, Cappagh National Orthopaedic Hospital, Finglas, Dublin 13, Ireland

## Abstract

**Background:**

Long term survivorship in total knee arthroplasty is significantly dependant on prosthesis alignment. Our aim was determine which alignment guide was more accurate in positioning of the tibial component in total knee arthroplasty. We also aimed to assess whether there was any difference in short term patient outcome.

**Method:**

A comparison of intramedullary versus extramedullary alignment jig was performed. Radiological alignment of tibial components and patient outcomes of 103 Triathlon total knee arthroplasties were analysed.

**Results:**

Use of the intramedullary was found to be significantly more accurate in determining coronal alignment (p = 0.02) while use of the extramedullary jig was found to give more accurate results in sagittal alignment (p = 0.04). There was no significant difference in WOMAC or SF-36 at six months.

**Conclusion:**

Use of an intramedullary jig is preferable for positioning of the tibial component using this knee system.

## Introduction

Long term survivorship in total knee arthroplasty is significantly dependant on prosthesis alignment. Several studies have correlated poor outcome with malalignment of the components [1]. Accuracy of component positioning relies on alignment guides for making precise and accurate bone cuts. Bargren et all reported a 91% failure rate for TKAs with varus tibio-femoral alignment and 11% of valgus alignment [2]. Significant contention still exists as to what the optimal alignment guide for placement of the tibial component is. While most patients are suitable for the use of either alignment system, patients with a large soft tissue envelope can preclude the use of an extramedullary guide while tibial deformity, previous fracture or retained metalwork can prevent use of an intramedullary guide.

Our aim was determine which alignment guide was more accurate in positioning of the tibial component in total knee arthroplasty. We also aimed to assess whether there was any difference in short term patient outcome.

## Materials & methods

We identified 103 consecutive total knee arthroplasties (TKA) in 95 patients which were performed between January 2008 and October 2008 by four Orthopaedic surgeons. The underlying diagnosis in all cases was primary osteoarthritis. There were no cases of tibial deformity precluding the use of an intramedullary jig. All patients underwent cemented total knee arthroplasty using the Triathlon knee system [Stryker, Kalamazoo, MI, USA]. The majority of patients (85 knees) had a posterior stabilised prosthesis with the remainder having a cruciate retaining design. This was performed under spinal anaesthesia using an above knee tourniquet. A medial parapatellar approach was used in all cases. Femoral alignment was determined using an intramedullary jig. In determining tibial alignment, an extramedullary or intramedullary alignment jig according to surgeon preference. If an extramedullary jig was used, a tibial cutting block with a posterior slope of 3° was used whereas if an intramedullary jig was used, the posterior slope was set at 0°. All patients were rehabilitated according to a standardised protocol.

Informed consent was obtained from all patients preoperatively to obtain data for the Cappagh National Orthopaedic hospital joint registry. Demographic data including age, gender and BMI was captured preoperatively. Operative details were captured at time of surgery. The WOMAC score was used as disease specific outcome score and the SF-36 was used as a general health outcome measure. These were captured preoperatively and at 6 months.

All patients had an AP and Lateral standing knee radiograph performed at 6 months. Coronal and Sagittal alignment of the tibial components were determined by an assessor blinded to which alignment jig was used intraoperatively. Three measurements were performed and a mean value for alignment was determined. Axis was measured in terms of deviation from the mechanical axis of the tibia.

Patient outcome data was captured using Bluespiers [Worcestershire, UK] clinical software. Information was collated using Microsoft Excel and statistical analysis was performed using Minitab statistical software http://www.minitab.com. A p value > 0.05 was taken as statistical significance.

## Results

There were 103 TKAs in total. In 36 cases, an intramedullary jig was used. There was no statistical difference between the two groups in terms of age, gender, body mass index (BMI) or length of inpatient hospital stay (LOS) [Table 1]. There were no complications associated with the use of an intramedullary jig.

**Table 1:** Patient Demographics

	Intra-medullary	Extra-medullary
Total TKA	36	67
Mean age	68.9	68.9
% male	21%	34%
Mean BMI	31.9	31.6
Mean LOS	10.5	9.1

**Figure 1:**
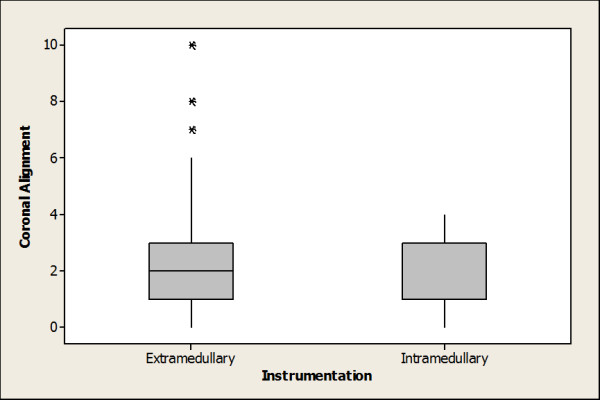
**Coronal Alignment**.

The mean coronal alignment of the tibial components in the intramedullary group was 1.6° from the mechanical axis and the mean coronal alignment in the extramedullary group was 2.4° [Table 2]. This was a statistically significant difference (p = 0.02). All patients in the intramedullary group were within two standard deviations of the mean alignment, while in the extramedullary group there were a number of outliers [Figure 1].

**Table 2:** Tibial Component Alignment

Coronal Alignment		p =
Intramedullary	1.6	0.02
Extramedullary	2.4	
**Sagittal Alignment**		
Intramedullary	3.4	0.07
Extramedullary	4.5	
**Sagittal Corrected**		
Intramedullary	3.1	0.04
Extramedullary	1.5	

**Figure 2:**
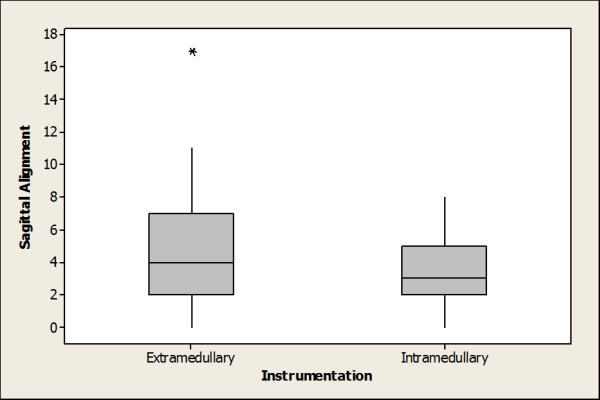
**Sagittal Alignment**.

The mean sagittal alignment of the tibial components in the intramedullary group was 3.4° from the mechanical axis and the mean sagittal alignment in the extramedullary group was 4.5°. This difference was not statistically different (p = 0.07). There were more outliers in the extramedullary group [Figure 2]. When the sagittal alignment measurement was corrected for the cutting jig used, the extramedullary jig was found to be within 1.5° of the intended 3° cut. This was significantly more accurate than the intramedullary jig (p = 0.04).

WOMAC and SF-36 scores were determined preoperatively and postoperatively [Table 3]. There was no significant difference in the preoperative WOMAC and SF-36 scores between the two groups. Postoperatively, the WOMAC score in the intramedullary group improved by 11.8 and the SF-36 by 12.7 while the improvement in the extramedullary group was 22.5 in both scores. This difference, however, did not reach significance (p = 0.06).

**Table 3:** Patient Outcome Scores

	Intra-medullary	Extra-medullary	p =
Pre WOMAC	43.2	45.9	0.6
6 mnth WOMAC	31.4	23.4	0.06
Change	11.8	22.5	
Pre SF-36	39.4	39.2	0.9
6 mnth SF-36	52.1	61.7	0.06
Change	-12.7	-22.5	

## Discussion

This study examined the tibial component alignment in similar groups of patients, in terms of patient demographics, who were treated by a group of four surgeons. The intramedullary guide was found to be more reliable for determining coronal alignment. Use of the extramedullary guide seemed to more reliably cut the desired posterior slope but the difference was only one degree. Regardless of which alignment jig was used, this did not seem to influence patient outcome.

Component alignment has been shown to have a bearing on patient outcome parameters. When analysing alignment parameters such as sagittal femoral, coronal femoral, rotational femoral, sagittal tibial, coronal tibial and femuro-tibial mismatch, this group found that when the number of alignment errors were reduced that the short term patient outcomes were significantly improved [3]. Use of the intramedullary jig seems to reduce the chance of outliers. This is one of the proposed benefits of navigated TKA [4]. However, computer navigated total knee arthroplasty has been found not to be a cost effective investment in terms of reducing revision risk in TKA [5].

A study of British orthopaedic surgeons found that 75.6% prefer extramedullary and 20.3% prefer intramedullary jigs when determining tibial alignment with the remainder using both or neither [6]. The published literature is divided as to which jig is superior. Rottman et al found no difference in alignment between intra- and extramedullary alignment in TKA in a retrospective series of 55 patients [7]. Reed et al performed a randomised prospective trial which showed that intramedullary guides were superior to extramedullary guides in determining coronal alignment of the tibial component [8]. In this study, we also found that the intramedullary guide was more reliable in determining coronal alignment. The mean deviation from the mechanical axis was 1.6 degrees with this jig but more importantly, there were no outliers.

There are relative indications for each method of alignment determination. Lozano et al examined obese patients and found no difference in the alignment of the tibial component between intra and extramedullary guides. However, there was a reduced tourniquet time associated with the intramedullary guide [9]. However, transesophageal echocardiography during the course of conventional intramedullary instrumented total knee procedures has demonstrated showers of fat or intramedullary embolic particles enter the right atrium of the heart in repeated and unpredictable patterns [10]. Most often these are clinically unimportant. Patients with significant extra-articular deformities, marked bowing, and those with prior surgery or fractures may not be suitable for intramedullary guides, and they may require the use of extramedullary guides and intra-operative radiographic control [11].

## Conclusions

This study has shown that use of an intramedullary alignment jig is more accurate in positioning the tibial component in TKA in terms of coronal alignment. Use of the extramedullary jig was found to be more accurate in terms of sagittal alignment. There was no significant difference in short term patient outcome scores. We would advocate the use of the intramedullary alignment jig to optimise tibial component positioning.

## Competing interests

The authors declare that they have no competing interests.

## Authors' contributions

JC participated in the design of the study and performed the statistical analysis. FC collected the study data and edited the manscript. KS and PK contributed to the study design and drafted the manuscript. All authors read and approved the final manuscript.
